# Supplementary material for: The Cellular Prion Protein Increases the Uptake and Toxicity of TDP-43 Fibrils
Source: Viruses. 2021 Aug 17;13(8):1625. doi: 10.3390/v13081625 (PMC8402629; doi:10.3390/v13081625)
Supplement: Supplementary file 1 [file viruses-13-01625-s001.zip › viruses-1327884-supplementary.pdf]

Supplementary Material

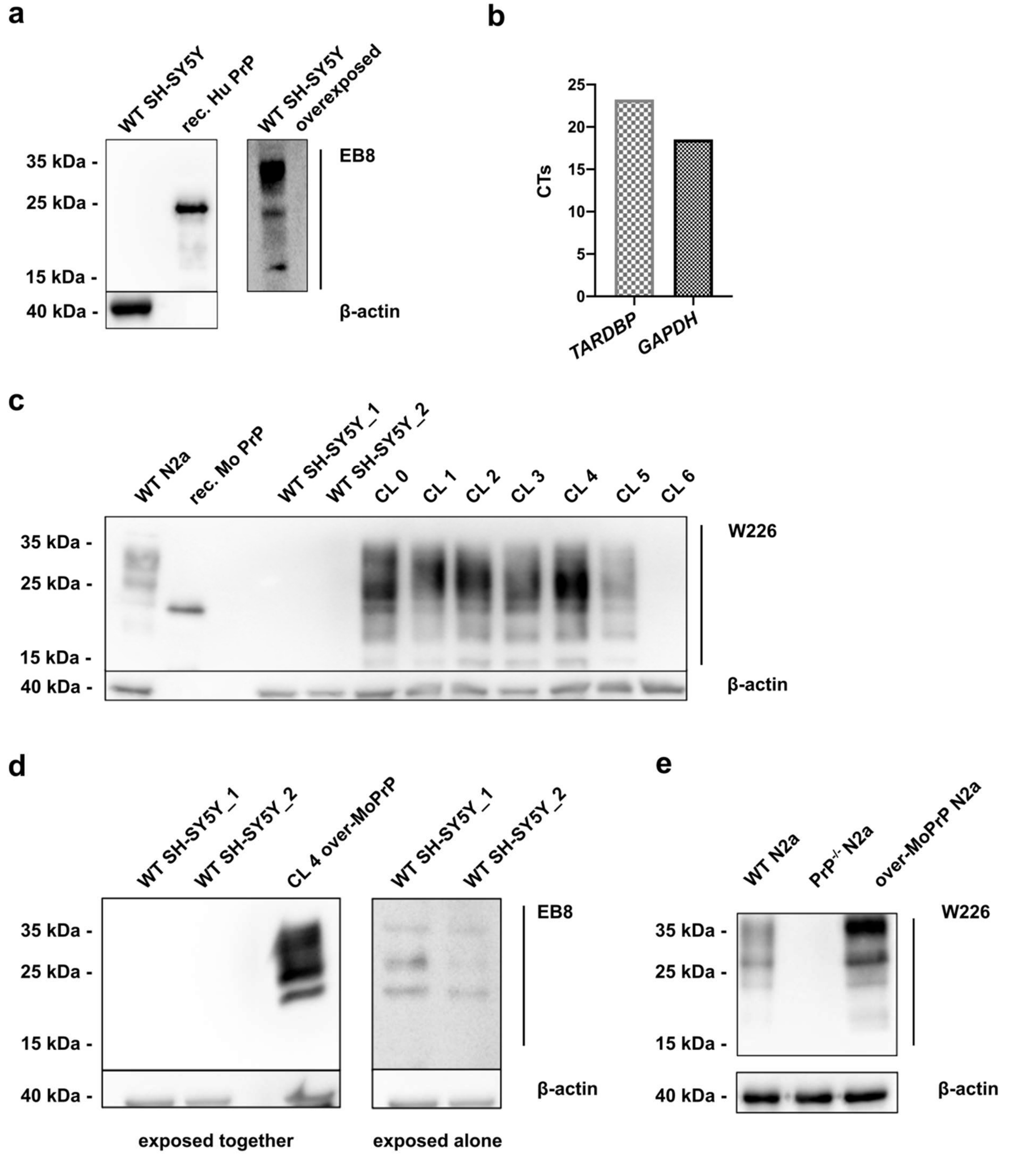

Figure S1. Expression levels of endogenous TDP-43 and PrP^{C} in WT SH-SY5Y cells, generation of a MoPrP^{C}-overexpressing cell line and verification of PrP$^{-/-}$ and over-MoPrP^{C} N2a cells. (a) WB analysis of WT-SHSY5Y cells showing the levels of expression of endogenous PrP^{C}; mouse recombinant PrP^{C} (recMoPrP) (25 ng) was loaded as positive control; after quantification 50 μg of total proteins were loaded for each cell lysate and β-actin was used as a loading control. Only when the membrane was cut and overexposed it was possible to observe a signal corresponding to the endogenous PrP^{C}; **(b)** real-time quantitative polymerase chain reaction (RT-qPCR) analysis of the expression of endogenous TDP-43 (*TARDBP*) in WT SH-SY5Y cells compared to the Glyceraldehyde-3-phosphate dehydrogenase (*GAPDH*) house-keeping gene. CTs: cycle thresholds. The evaluation was performed in triplicate; **(c)** generation of MoPrP^{C} overexpressing SH-SY5Y cells and selection of the most overexpressing clone (CL 4) by means of WB using the anti-PrP W226 antibody; recMoPrP (25 ng) was loaded as positive control; **(d)** comparison of MoPrP^{C} expression levels between over-MoPrP SH-SY5Y cells and two different samples of WT SH-SY5Y cell lysates; **(e)**

comparison of MoPrP^{C} expression levels between WT, PrP$^{-/-}$ and over-MoPrP N2a cells. After quantification 50 μg of total proteins were loaded for each cell lysate and β-actin was used as a loading control.

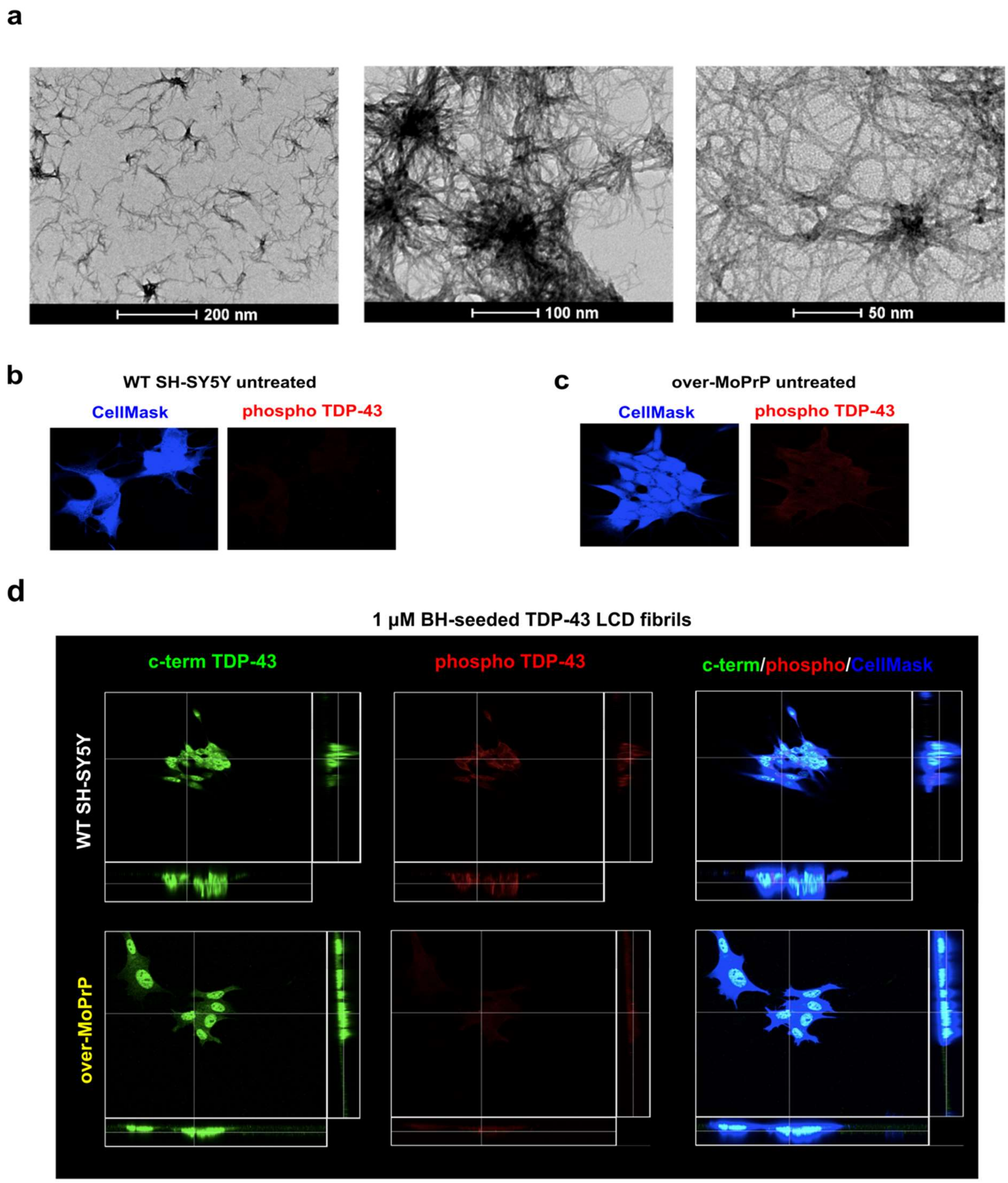

Figure S2. Intracellular uptake and phosphorylation of BH-seeded TDP-43 LCD fibrils. **(a)** Transmission electron microscopy analysis of TDP-43 LCD BH-seeded fibrils collected when the reaction reached its plateau; representative immunofluorescence images of untreated WT **(b)** and over-MoPrP **(c)** SH-SY5Y cells; blue staining corresponds to the CellMask which defines whole-cell bodies while the red staining corresponds to the anti-phospho TDP-43 (Ser409/Ser410) antibody; **(d)** representative orthogonal views of the 3D Z-stack for WT and over-MoPrP cells treated with 1 μM BH-seeded TDP-43 LCD fibrils following 6 hours of incubation and 24 hours before analysis; since BH-seeded fibrils were not conjugated with any fluorescent dye**,** the green staining corresponds to the signal of the anti-TDP-43 C-terminal antibody which recognizes both endogenous TDP-43 (mainly nuclear) and the *in vitro* produced TDP-43 LCD fibrils. Red spots correspond to TDP-43 aggregates recognized by the anti-phospho TDP-43 (Ser409/Ser410) antibody; all experiments represented in the image were replicated three times.
